# {6,6′-Dieth­oxy-2,2′-[2,2-dimethyl­propane-1,3-diylbis(nitrilo­methyl­idyne)]diphenolato}nickel(II) monohydrate

**DOI:** 10.1107/S1600536809008721

**Published:** 2009-03-14

**Authors:** Hadi Kargar, Arezoo Jamshidvand, Hoong-Kun Fun, Reza Kia

**Affiliations:** a Department of Chemistry, School of Science, Payame Noor University (PNU), Ardakan, Yazd, Iran; b X-ray Crystallography Unit, School of Physics, Universiti Sains Malaysia, 11800 USM, Penang, Malaysia

## Abstract

In the title complex, [Ni(C_23_H_28_N_2_O_4_)]·H_2_O, the Ni^II^ ion is coordinated by the N_2_O_2_ unit of the tetra­dentate Schiff base ligand in a slightly distorted planar geometry. The asymmetric unit of the title compound comprises one complex mol­ecule and a water mol­ecule of crystallization. The H atoms of the water mol­ecule make bifurcated inter­molecular hydrogen bonds with the O atoms of the phenolate and eth­oxy groups with *R*_1_^2^(5) and *R*_1_^2^(6) ring motifs, which may, in part, influence the mol­ecular configuration. The dihedral angle between the two benzene rings is 31.43 (5)°. The crystal structure is further stabilized by inter­molecular C—H⋯O and C—H⋯π inter­actions, which link neighbouring mol­ecules into one-dimensional extended chains along the *a* axis. An inter­esting feature of the crystal structure is the short inter­molecular C⋯C [3.3044 (14) Å] contact which is shorter than the sum of the van der Waals radii.

## Related literature

For bond-length data, see Allen *et al.* (1987 ▶). For related structures see, for example: Clark *et al.* (1968 ▶, 1969 ▶, 1970 ▶). For the applications and bioactivities of Schiff base complexes with transition metals, see, for example: Elmali *et al.* (2000 ▶); Blower (1998 ▶); Granovski *et al.*, (1993 ▶); Li & Chang (1991 ▶); Shahrokhian *et al.* (2000 ▶). For the stability of the temperature controller used for the data collection, see: Cosier & Glazer (1986 ▶). For hydrogen-bond motifs, see: Bernstein *et al.* (1995 ▶).
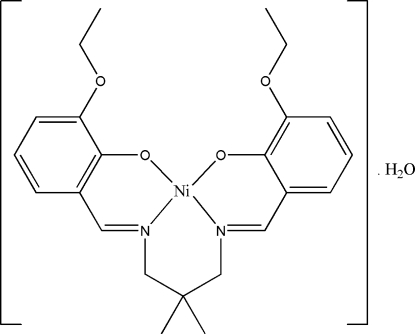


## Experimental

### 

#### Crystal data

[Ni(C_23_H_28_N_2_O_4_)]·H_2_O
*M* *_r_* = 473.20
Triclinic, 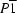

*a* = 9.3797 (1) Å
*b* = 10.7570 (1) Å
*c* = 12.8002 (1) Å
α = 65.811 (1)°
β = 68.87°
γ = 78.336 (1)°
*V* = 1096.64 (2) Å^3^
*Z* = 2
Mo *K*α radiation
μ = 0.92 mm^−1^
*T* = 100 K
0.36 × 0.19 × 0.14 mm

#### Data collection

Bruker SMART APEXII CCD area-detector diffractometer
Absorption correction: multi-scan (**SADABS**; Bruker, 2005 ▶) *T*_min_ = 0.733, *T*_max_ = 0.879
29688 measured reflections
9211 independent reflections
8270 reflections with *I* > 2σ*I*)
*R*_int_ = 0.023

#### Refinement

*R*[*F* ^2^ > 2σ(*F* ^2^)] = 0.027
*wR*(*F* ^2^) = 0.073
*S* = 1.04
9211 reflections
290 parameters
H atoms treated by a mixture of independent and constrained refinement
Δρ_max_ = 0.51 e Å^−3^
Δρ_min_ = −0.54 e Å^−3^

### 

Data collection: *APEX2* (Bruker, 2005 ▶); cell refinement: *SAINT* (Bruker, 2005 ▶); data reduction: *SAINT*; program(s) used to solve structure: *SHELXTL* (Sheldrick, 2008 ▶); program(s) used to refine structure: *SHELXTL*; molecular graphics: *SHELXTL*; software used to prepare material for publication: *SHELXTL* and *PLATON* (Spek, 2009 ▶).

## Supplementary Material

Crystal structure: contains datablocks global, I. DOI: 10.1107/S1600536809008721/at2741sup1.cif

Structure factors: contains datablocks I. DOI: 10.1107/S1600536809008721/at2741Isup2.hkl

Additional supplementary materials:  crystallographic information; 3D view; checkCIF report

## Figures and Tables

**Table 1:** Selected bond lengths (Å)

Ni1—O1	1.8523 (6)
Ni1—O2	1.8605 (6)
Ni1—N2	1.8748 (7)
Ni1—N1	1.8766 (8)

**Table 2:** Hydrogen-bond geometry (Å, °)

*D*—H⋯*A*	*D*—H	H⋯*A*	*D*⋯*A*	*D*—H⋯*A*
O1*W*—H2*W*1⋯O1	0.80 (2)	2.499 (19)	3.0368 (12)	125.8 (16)
O1*W*—H2*W*1⋯O3	0.80 (2)	2.20 (2)	2.9805 (12)	164.7 (18)
O1*W*—H1*W*1⋯O2	0.77 (2)	2.15 (2)	2.8597 (10)	152 (2)
O1*W*—H1*W*1⋯O4	0.77 (2)	2.53 (2)	3.1658 (12)	140 (2)
C8—H8*B*⋯O1^i^	0.99	2.42	3.3216 (13)	151
C11—H11*A*⋯O1*W*^ii^	0.95	2.51	3.4242 (13)	161
C5—-H5*A*⋯*Cg*1^i^	0.95	2.88	3.3506 (12)	111
C10—-H10*B*⋯*Cg*1^ii^	0.99	2.73	3.4406 (11)	129
C22—-H22*B*⋯*Cg*2^iii^	0.99	2.87	3.8068 (11)	158

Symmetry codes: (i) 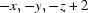; (ii) 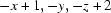; (iii) 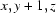. *Cg*1 and *Cg*2 are the centroids of the C12–C17 and C1–C6 benzene rings, respectively.

**Fig. 1.**
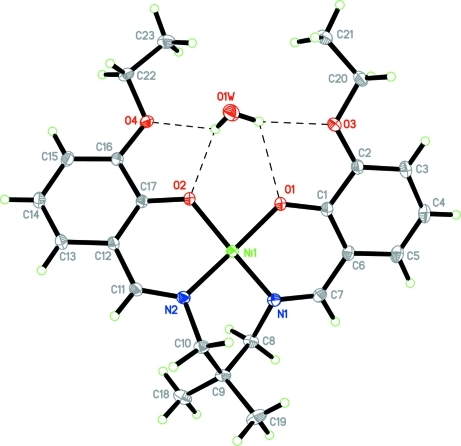
The molecular structure of the title compound, showing 50% probability displacement ellipsoids and the atomic numbering. Intermolecular hydrogen bonds are shown as dashed lines.

**Fig. 2.**
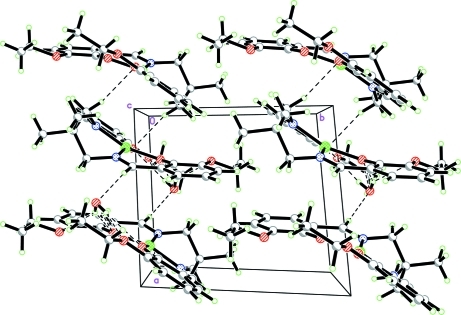
The crystal packing of the title compound viewed down the c-axis, showing 1-D extended chains along the a-axis. Intermolecular interactions are drawn as dashed lines.

## supplementary crystallographic information

### Comment

Schiff base complexes are some of the most important stereochemical models in transition metal coordination chemistry, with their ease of preparation and structural variations (Granovski *et al.*, 1993). Metal derivatives of Schiff bases have been studied extensively, and copper(II) and Ni(II) complexes play a major role in both synthetic and structural research (Elmali *et al.*, 2000; Blower, 1998; Granovski *et al.*, 1993; Li & Chang, 1991; Shahrokhian *et al.*, 2000). Tetradentate Schiff base metal complexes may form *trans* or *cis* planar or tetrahedral structures (Elmali *et al.*, 2000).

The Ni^II^ ion of the title compound (Fig. 1), shows a sligthly distorted planar geometry which is coordinated by two imine N atoms and two phenol O atoms of the tetradentate Schiff base ligand. The bond lengths (Allen *et al.*, 1987) and angles are within normal ranges and are comparable with the related structures (Clark *et al.*, 1968, 1969, 1970). The asymmetric unit of the title compound comprises one molecule of complex and a water molecule of crystallization. The hydrogen atoms of the water molecule make bifurcated intermolecular hydrogen bonds with the oxygen atoms of the phenolato- and ethoxy groups with *R^2^_1_(5)* and *R^2^_1_(6)* ring motifs (Bernstein *et al.*, 1995), which may, in part, influence the molecular configuration. The dihedral angle between the two benzene rings is 31.43 (5)°. The crystal structure is further stabilized by intermolecular C—H···O and C—H···π interactions which link neighbouring molecules into 1-D extended chains along the *a* axis. The interesting feature of the crystal structure is a short intermolecular C7···C17^i^ [3.3044 (14) Å] contact which is shorter than the sum of the van der Waals radius of a carbon atom. The crystal structure is further stabilized by intermolecular C—H···O and C—H···π interactions (Table 2, *Cg*1 and *Cg*2 are the centroids of the C12–C17 and C1–C6 benzene rings) which link neighbouring molecules into 1-D extended chains along the *b*-axis (Fig. 2).

### Experimental

A chloroform solution (40 ml) of [*N,N'*-Bis(3-ethoxy-salicylidene)-2, 2-dimethyl-1,3-propanediamin (1 mmol) was added to a ethanol solution (20 mL) of NiCl_2_.6H_2_O (1.05 mmol, 237 mg). The mixture was refluxed for 30 min and then filtered. After keeping the filtrate in air, deep-green block-shaped crystals were formed at the bottom of the vessel on slow evaporation of the solvent.

### Refinement

The water H-atoms were located from the difference Fourier map and freely refined. The rest of the hydrogen atoms were positioned geometrically [C—H = 0.95–99 Å] and refined using a riding approximation model with U_iso_(H) = 1.2 or 1.5U_eq_(C). A rotating-group model was used for the methyl groups of the ethoxy substituents.

### Crystal data

**Table:**

[Ni(C_23_H_28_N_2_O_4_)]·H_2_O	*Z* = 2
*M**_r_* = 473.20	*F*(000) = 500
Triclinic, *P*1	*D*_x_ = 1.433 Mg m^−^^3^
Hall symbol: -P 1	Mo *K*α radiation, λ = 0.71073 Å
*a* = 9.3797 (1) Å	Cell parameters from 9961 reflections
*b* = 10.7570 (1) Å	θ = 2.2–39.8°
*c* = 12.8002 (1) Å	µ = 0.92 mm^−^^1^
α = 65.811 (1)°	*T* = 100 K
β = 68.87°	Block, green
γ = 78.336 (1)°	0.36 × 0.19 × 0.14 mm
*V* = 1096.64 (2) Å^3^	

### Data collection

**Table:**

Bruker SMART APEXII CCD area-detector diffractometer	8270 reflections with *I* > 2σ(*I*)
φ and ω scans	*R*_int_ = 0.023
Absorption correction: multi-scan (*SADABS*; Bruker, 2005)	θ_max_ = 34.5°, θ_min_ = 1.8°
*T*_min_ = 0.733, *T*_max_ = 0.879	*h* = −14→14
29688 measured reflections	*k* = −17→16
9211 independent reflections	*l* = −20→20

### Refinement

**Table:**

Refinement on *F*^2^	Primary atom site location: structure-invariant direct methods
Least-squares matrix: full	Secondary atom site location: difference Fourier map
*R*[*F*^2^ > 2σ(*F*^2^)] = 0.027	Hydrogen site location: inferred from neighbouring sites
*wR*(*F*^2^) = 0.073	H atoms treated by a mixture of independent and constrained refinement
*S* = 1.04	*w* = 1/[σ^2^(*F*_o_^2^) + (0.0345*P*)^2^ + 0.3434*P*] where *P* = (*F*_o_^2^ + 2*F*_c_^2^)/3
9211 reflections	(Δ/σ)_max_ = 0.002
290 parameters	Δρ_max_ = 0.51 e Å^−^^3^
0 restraints	Δρ_min_ = −0.54 e Å^−^^3^

### Special details

**Table:**

Experimental. The crystal was placed in the cold stream of an Oxford Cyrosystems Cobra open-flow nitrogen cryostat (Cosier & Glazer, 1986) operating at 100.0 (1)K.
Geometry. All esds (except the esd in the dihedral angle between two l.s. planes) are estimated using the full covariance matrix. The cell esds are taken into account individually in the estimation of esds in distances, angles and torsion angles; correlations between esds in cell parameters are only used when they are defined by crystal symmetry. An approximate (isotropic) treatment of cell esds is used for estimating esds involving l.s. planes.
Refinement. Refinement of F^2^ against ALL reflections. The weighted R-factor wR and goodness of fit S are based on F^2^, conventional R-factors R are based on F, with F set to zero for negative F^2^. The threshold expression of F^2^ > 2sigma(F^2^) is used only for calculating R-factors(gt) etc. and is not relevant to the choice of reflections for refinement. R-factors based on F^2^ are statistically about twice as large as those based on F, and R- factors based on ALL data will be even larger.

### Fractional atomic coordinates and isotropic or equivalent isotropic displacement parameters (Å^2^)

**Table:**

	*x*	*y*	*z*	*U*_iso_*/*U*_eq_	
Ni1	0.226401 (12)	−0.053714 (11)	0.977253 (10)	0.01153 (3)	
O1	0.23133 (8)	−0.03160 (7)	0.82469 (6)	0.01499 (11)	
O2	0.29583 (8)	0.12152 (7)	0.90220 (6)	0.01433 (11)	
O3	0.28411 (8)	0.03631 (7)	0.59447 (6)	0.01764 (12)	
O4	0.30413 (8)	0.38398 (7)	0.78316 (6)	0.01683 (12)	
N1	0.10045 (8)	−0.20138 (8)	1.05666 (7)	0.01348 (12)	
N2	0.28140 (8)	−0.09129 (8)	1.11477 (7)	0.01317 (12)	
C1	0.17327 (10)	−0.10785 (9)	0.79462 (8)	0.01354 (14)	
C2	0.19469 (10)	−0.07175 (9)	0.66932 (8)	0.01518 (14)	
C3	0.12695 (11)	−0.14175 (10)	0.63155 (9)	0.01851 (16)	
H3A	0.1417	−0.1157	0.5481	0.022*	
C4	0.03610 (12)	−0.25156 (11)	0.71655 (10)	0.02041 (17)	
H4A	−0.0122	−0.2979	0.6905	0.024*	
C5	0.01751 (11)	−0.29140 (10)	0.83686 (9)	0.01839 (16)	
H5A	−0.0420	−0.3666	0.8936	0.022*	
C6	0.08636 (10)	−0.22137 (9)	0.87752 (8)	0.01478 (14)	
C7	0.05068 (10)	−0.25756 (9)	1.00486 (8)	0.01497 (14)	
H7A	−0.0157	−0.3295	1.0562	0.018*	
C8	0.02317 (10)	−0.23401 (9)	1.18710 (8)	0.01525 (15)	
H8A	−0.0543	−0.3007	1.2157	0.018*	
H8B	−0.0316	−0.1499	1.1996	0.018*	
C9	0.13208 (10)	−0.29300 (9)	1.26399 (8)	0.01469 (14)	
C10	0.29062 (10)	−0.23683 (9)	1.19059 (8)	0.01490 (14)	
H10A	0.3476	−0.2494	1.2463	0.018*	
H10B	0.3484	−0.2895	1.1387	0.018*	
C11	0.33388 (10)	−0.00677 (9)	1.13864 (8)	0.01485 (14)	
H11A	0.3686	−0.0425	1.2072	0.018*	
C12	0.34334 (10)	0.13722 (9)	1.06889 (8)	0.01425 (14)	
C13	0.38100 (12)	0.22038 (10)	1.11540 (9)	0.01869 (16)	
H13A	0.4020	0.1802	1.1901	0.022*	
C14	0.38748 (12)	0.35894 (10)	1.05349 (9)	0.01931 (17)	
H14A	0.4100	0.4146	1.0864	0.023*	
C15	0.36063 (11)	0.41821 (10)	0.94107 (9)	0.01667 (15)	
H15A	0.3634	0.5142	0.8989	0.020*	
C16	0.33023 (10)	0.33740 (9)	0.89181 (8)	0.01361 (14)	
C17	0.32095 (9)	0.19304 (9)	0.95500 (8)	0.01256 (13)	
C18	0.06211 (12)	−0.25176 (11)	1.37441 (9)	0.02151 (18)	
H18A	0.0491	−0.1520	1.3479	0.032*	
H18B	0.1303	−0.2864	1.4244	0.032*	
H18C	−0.0378	−0.2906	1.4215	0.032*	
C19	0.15271 (12)	−0.44884 (10)	1.30393 (11)	0.02356 (19)	
H19A	0.2224	−0.4841	1.3527	0.035*	
H19B	0.1958	−0.4741	1.2328	0.035*	
H19C	0.0532	−0.4880	1.3519	0.035*	
C20	0.29788 (12)	0.09025 (10)	0.46874 (8)	0.01952 (17)	
H20A	0.1955	0.1167	0.4575	0.023*	
H20B	0.3498	0.0213	0.4322	0.023*	
C21	0.39176 (14)	0.21404 (11)	0.41110 (9)	0.02295 (19)	
H21A	0.3962	0.2597	0.3263	0.034*	
H21B	0.4957	0.1851	0.4164	0.034*	
H21C	0.3440	0.2774	0.4533	0.034*	
C22	0.35841 (11)	0.51438 (9)	0.69639 (9)	0.01766 (16)	
H22A	0.4706	0.5140	0.6771	0.021*	
H22B	0.3085	0.5873	0.7284	0.021*	
C23	0.31822 (12)	0.53790 (11)	0.58548 (9)	0.02132 (18)	
H23A	0.3592	0.6233	0.5216	0.032*	
H23B	0.2066	0.5439	0.6047	0.032*	
H23C	0.3627	0.4618	0.5580	0.032*	
O1W	0.47217 (9)	0.17324 (9)	0.65560 (7)	0.02297 (15)	
H2W1	0.422 (2)	0.1245 (18)	0.6513 (16)	0.037 (5)*	
H1W1	0.419 (2)	0.187 (2)	0.7124 (19)	0.051 (6)*	

### Atomic displacement parameters (Å^2^)

**Table:**

	*U*^11^	*U*^22^	*U*^33^	*U*^12^	*U*^13^	*U*^23^
Ni1	0.01281 (5)	0.01131 (5)	0.01095 (5)	−0.00182 (3)	−0.00487 (4)	−0.00308 (4)
O1	0.0199 (3)	0.0142 (3)	0.0134 (3)	−0.0042 (2)	−0.0070 (2)	−0.0047 (2)
O2	0.0197 (3)	0.0125 (3)	0.0130 (3)	−0.0036 (2)	−0.0068 (2)	−0.0040 (2)
O3	0.0247 (3)	0.0172 (3)	0.0125 (3)	−0.0044 (2)	−0.0076 (2)	−0.0040 (2)
O4	0.0215 (3)	0.0138 (3)	0.0151 (3)	−0.0060 (2)	−0.0086 (2)	−0.0004 (2)
N1	0.0123 (3)	0.0136 (3)	0.0136 (3)	−0.0012 (2)	−0.0045 (2)	−0.0036 (2)
N2	0.0133 (3)	0.0135 (3)	0.0122 (3)	−0.0014 (2)	−0.0051 (2)	−0.0031 (2)
C1	0.0141 (3)	0.0130 (3)	0.0159 (4)	0.0007 (3)	−0.0066 (3)	−0.0067 (3)
C2	0.0171 (4)	0.0149 (4)	0.0162 (4)	0.0001 (3)	−0.0071 (3)	−0.0071 (3)
C3	0.0209 (4)	0.0207 (4)	0.0199 (4)	−0.0005 (3)	−0.0089 (3)	−0.0114 (3)
C4	0.0200 (4)	0.0232 (4)	0.0258 (5)	−0.0024 (3)	−0.0084 (3)	−0.0148 (4)
C5	0.0160 (4)	0.0195 (4)	0.0229 (4)	−0.0038 (3)	−0.0045 (3)	−0.0110 (3)
C6	0.0133 (3)	0.0153 (4)	0.0174 (4)	−0.0015 (3)	−0.0045 (3)	−0.0076 (3)
C7	0.0125 (3)	0.0146 (4)	0.0174 (4)	−0.0020 (3)	−0.0043 (3)	−0.0052 (3)
C8	0.0115 (3)	0.0181 (4)	0.0134 (3)	−0.0008 (3)	−0.0033 (3)	−0.0037 (3)
C9	0.0132 (3)	0.0137 (3)	0.0136 (3)	−0.0005 (3)	−0.0043 (3)	−0.0017 (3)
C10	0.0132 (3)	0.0140 (3)	0.0154 (4)	0.0001 (3)	−0.0061 (3)	−0.0025 (3)
C11	0.0152 (3)	0.0172 (4)	0.0125 (3)	−0.0024 (3)	−0.0055 (3)	−0.0041 (3)
C12	0.0155 (3)	0.0164 (4)	0.0124 (3)	−0.0036 (3)	−0.0049 (3)	−0.0052 (3)
C13	0.0228 (4)	0.0221 (4)	0.0152 (4)	−0.0062 (3)	−0.0070 (3)	−0.0076 (3)
C14	0.0222 (4)	0.0212 (4)	0.0189 (4)	−0.0068 (3)	−0.0057 (3)	−0.0096 (3)
C15	0.0167 (4)	0.0163 (4)	0.0183 (4)	−0.0042 (3)	−0.0044 (3)	−0.0072 (3)
C16	0.0126 (3)	0.0145 (3)	0.0140 (3)	−0.0025 (3)	−0.0043 (3)	−0.0047 (3)
C17	0.0117 (3)	0.0138 (3)	0.0130 (3)	−0.0021 (2)	−0.0039 (3)	−0.0052 (3)
C18	0.0204 (4)	0.0266 (5)	0.0143 (4)	0.0003 (3)	−0.0051 (3)	−0.0056 (3)
C19	0.0205 (4)	0.0134 (4)	0.0302 (5)	−0.0011 (3)	−0.0082 (4)	−0.0014 (4)
C20	0.0286 (5)	0.0188 (4)	0.0137 (4)	−0.0003 (3)	−0.0089 (3)	−0.0069 (3)
C21	0.0347 (5)	0.0193 (4)	0.0141 (4)	−0.0035 (4)	−0.0074 (4)	−0.0048 (3)
C22	0.0174 (4)	0.0138 (4)	0.0185 (4)	−0.0039 (3)	−0.0065 (3)	−0.0006 (3)
C23	0.0216 (4)	0.0201 (4)	0.0175 (4)	−0.0021 (3)	−0.0070 (3)	−0.0013 (3)
O1W	0.0231 (3)	0.0296 (4)	0.0172 (3)	−0.0094 (3)	−0.0036 (3)	−0.0084 (3)

### Geometric parameters (Å, °)

**Table:**

Ni1—O1	1.8523 (6)	C10—H10B	0.9900
Ni1—O2	1.8605 (6)	C11—C12	1.4385 (12)
Ni1—N2	1.8748 (7)	C11—H11A	0.9500
Ni1—N1	1.8766 (8)	C12—C17	1.4079 (12)
O1—C1	1.3068 (10)	C12—C13	1.4127 (12)
O2—C17	1.3109 (10)	C13—C14	1.3738 (14)
O3—C2	1.3692 (11)	C13—H13A	0.9500
O3—C20	1.4349 (11)	C14—C15	1.4089 (14)
O4—C16	1.3675 (11)	C14—H14A	0.9500
O4—C22	1.4370 (11)	C15—C16	1.3833 (12)
N1—C7	1.2994 (11)	C15—H15A	0.9500
N1—C8	1.4786 (12)	C16—C17	1.4310 (12)
N2—C11	1.2960 (11)	C18—H18A	0.9800
N2—C10	1.4700 (11)	C18—H18B	0.9800
C1—C6	1.4137 (12)	C18—H18C	0.9800
C1—C2	1.4327 (12)	C19—H19A	0.9800
C2—C3	1.3848 (12)	C19—H19B	0.9800
C3—C4	1.4109 (15)	C19—H19C	0.9800
C3—H3A	0.9500	C20—C21	1.5144 (15)
C4—C5	1.3716 (14)	C20—H20A	0.9900
C4—H4A	0.9500	C20—H20B	0.9900
C5—C6	1.4197 (13)	C21—H21A	0.9800
C5—H5A	0.9500	C21—H21B	0.9800
C6—C7	1.4352 (13)	C21—H21C	0.9800
C7—H7A	0.9500	C22—C23	1.5081 (14)
C8—C9	1.5382 (12)	C22—H22A	0.9900
C8—H8A	0.9900	C22—H22B	0.9900
C8—H8B	0.9900	C23—H23A	0.9800
C9—C19	1.5320 (13)	C23—H23B	0.9800
C9—C10	1.5330 (12)	C23—H23C	0.9800
C9—C18	1.5337 (14)	O1W—H2W1	0.798 (19)
C10—H10A	0.9900	O1W—H1W1	0.77 (2)
			
O1—Ni1—O2	84.55 (3)	C12—C11—H11A	117.6
O1—Ni1—N2	163.37 (3)	C17—C12—C13	120.81 (8)
O2—Ni1—N2	93.63 (3)	C17—C12—C11	120.71 (8)
O1—Ni1—N1	94.46 (3)	C13—C12—C11	118.45 (8)
O2—Ni1—N1	163.13 (3)	C14—C13—C12	120.52 (8)
N2—Ni1—N1	91.99 (3)	C14—C13—H13A	119.7
C1—O1—Ni1	128.09 (6)	C12—C13—H13A	119.7
C17—O2—Ni1	126.23 (6)	C13—C14—C15	119.80 (8)
C2—O3—C20	118.18 (7)	C13—C14—H14A	120.1
C16—O4—C22	117.93 (7)	C15—C14—H14A	120.1
C7—N1—C8	116.61 (7)	C16—C15—C14	120.37 (9)
C7—N1—Ni1	125.70 (6)	C16—C15—H15A	119.8
C8—N1—Ni1	116.04 (6)	C14—C15—H15A	119.8
C11—N2—C10	117.40 (7)	O4—C16—C15	125.18 (8)
C11—N2—Ni1	126.74 (6)	O4—C16—C17	113.82 (7)
C10—N2—Ni1	114.92 (6)	C15—C16—C17	120.99 (8)
O1—C1—C6	124.54 (8)	O2—C17—C12	124.49 (8)
O1—C1—C2	117.97 (8)	O2—C17—C16	118.10 (7)
C6—C1—C2	117.46 (8)	C12—C17—C16	117.39 (8)
O3—C2—C3	124.85 (8)	C9—C18—H18A	109.5
O3—C2—C1	114.03 (8)	C9—C18—H18B	109.5
C3—C2—C1	121.11 (9)	H18A—C18—H18B	109.5
C2—C3—C4	120.24 (9)	C9—C18—H18C	109.5
C2—C3—H3A	119.9	H18A—C18—H18C	109.5
C4—C3—H3A	119.9	H18B—C18—H18C	109.5
C5—C4—C3	120.01 (9)	C9—C19—H19A	109.5
C5—C4—H4A	120.0	C9—C19—H19B	109.5
C3—C4—H4A	120.0	H19A—C19—H19B	109.5
C4—C5—C6	120.67 (9)	C9—C19—H19C	109.5
C4—C5—H5A	119.7	H19A—C19—H19C	109.5
C6—C5—H5A	119.7	H19B—C19—H19C	109.5
C1—C6—C5	120.43 (8)	O3—C20—C21	106.38 (8)
C1—C6—C7	120.59 (8)	O3—C20—H20A	110.5
C5—C6—C7	118.55 (8)	C21—C20—H20A	110.5
N1—C7—C6	126.29 (8)	O3—C20—H20B	110.5
N1—C7—H7A	116.9	C21—C20—H20B	110.5
C6—C7—H7A	116.9	H20A—C20—H20B	108.6
N1—C8—C9	114.08 (7)	C20—C21—H21A	109.5
N1—C8—H8A	108.7	C20—C21—H21B	109.5
C9—C8—H8A	108.7	H21A—C21—H21B	109.5
N1—C8—H8B	108.7	C20—C21—H21C	109.5
C9—C8—H8B	108.7	H21A—C21—H21C	109.5
H8A—C8—H8B	107.6	H21B—C21—H21C	109.5
C19—C9—C10	107.48 (7)	O4—C22—C23	106.57 (8)
C19—C9—C18	110.13 (8)	O4—C22—H22A	110.4
C10—C9—C18	110.50 (8)	C23—C22—H22A	110.4
C19—C9—C8	110.92 (8)	O4—C22—H22B	110.4
C10—C9—C8	110.21 (7)	C23—C22—H22B	110.4
C18—C9—C8	107.62 (7)	H22A—C22—H22B	108.6
N2—C10—C9	112.31 (7)	C22—C23—H23A	109.5
N2—C10—H10A	109.1	C22—C23—H23B	109.5
C9—C10—H10A	109.1	H23A—C23—H23B	109.5
N2—C10—H10B	109.1	C22—C23—H23C	109.5
C9—C10—H10B	109.1	H23A—C23—H23C	109.5
H10A—C10—H10B	107.9	H23B—C23—H23C	109.5
N2—C11—C12	124.73 (8)	H2W1—O1W—H1W1	100.8 (18)
N2—C11—H11A	117.6		
			
O2—Ni1—O1—C1	−168.23 (8)	Ni1—N1—C7—C6	2.41 (13)
N2—Ni1—O1—C1	107.35 (12)	C1—C6—C7—N1	−5.56 (14)
N1—Ni1—O1—C1	−5.14 (8)	C5—C6—C7—N1	−178.10 (9)
O1—Ni1—O2—C17	177.64 (7)	C7—N1—C8—C9	124.62 (9)
N2—Ni1—O2—C17	−18.95 (7)	Ni1—N1—C8—C9	−69.17 (9)
N1—Ni1—O2—C17	90.23 (12)	N1—C8—C9—C19	−89.00 (9)
O1—Ni1—N1—C7	2.24 (8)	N1—C8—C9—C10	29.91 (11)
O2—Ni1—N1—C7	88.17 (13)	N1—C8—C9—C18	150.47 (8)
N2—Ni1—N1—C7	−162.42 (8)	C11—N2—C10—C9	115.54 (9)
O1—Ni1—N1—C8	−162.55 (6)	Ni1—N2—C10—C9	−74.85 (8)
O2—Ni1—N1—C8	−76.61 (12)	C19—C9—C10—N2	161.00 (8)
N2—Ni1—N1—C8	32.80 (6)	C18—C9—C10—N2	−78.80 (9)
O1—Ni1—N2—C11	88.87 (13)	C8—C9—C10—N2	40.01 (10)
O2—Ni1—N2—C11	5.77 (8)	C10—N2—C11—C12	175.62 (8)
N1—Ni1—N2—C11	−158.31 (8)	Ni1—N2—C11—C12	7.39 (13)
O1—Ni1—N2—C10	−79.61 (12)	N2—C11—C12—C17	−11.73 (14)
O2—Ni1—N2—C10	−162.71 (6)	N2—C11—C12—C13	170.51 (9)
N1—Ni1—N2—C10	33.21 (6)	C17—C12—C13—C14	3.93 (14)
Ni1—O1—C1—C6	3.45 (13)	C11—C12—C13—C14	−178.31 (9)
Ni1—O1—C1—C2	−178.65 (6)	C12—C13—C14—C15	−1.80 (15)
C20—O3—C2—C3	6.21 (13)	C13—C14—C15—C16	−1.05 (15)
C20—O3—C2—C1	−172.95 (8)	C22—O4—C16—C15	21.08 (13)
O1—C1—C2—O3	3.97 (12)	C22—O4—C16—C17	−159.98 (8)
C6—C1—C2—O3	−177.98 (8)	C14—C15—C16—O4	−179.31 (8)
O1—C1—C2—C3	−175.22 (8)	C14—C15—C16—C17	1.82 (14)
C6—C1—C2—C3	2.83 (13)	Ni1—O2—C17—C12	19.68 (12)
O3—C2—C3—C4	−179.69 (9)	Ni1—O2—C17—C16	−161.96 (6)
C1—C2—C3—C4	−0.59 (14)	C13—C12—C17—O2	175.28 (9)
C2—C3—C4—C5	−1.52 (15)	C11—C12—C17—O2	−2.42 (13)
C3—C4—C5—C6	1.30 (15)	C13—C12—C17—C16	−3.09 (13)
O1—C1—C6—C5	174.88 (8)	C11—C12—C17—C16	179.21 (8)
C2—C1—C6—C5	−3.04 (13)	O4—C16—C17—O2	2.78 (11)
O1—C1—C6—C7	2.48 (14)	C15—C16—C17—O2	−178.23 (8)
C2—C1—C6—C7	−175.44 (8)	O4—C16—C17—C12	−178.75 (8)
C4—C5—C6—C1	1.04 (14)	C15—C16—C17—C12	0.25 (12)
C4—C5—C6—C7	173.59 (9)	C2—O3—C20—C21	175.61 (8)
C8—N1—C7—C6	167.12 (8)	C16—O4—C22—C23	178.53 (8)

### Hydrogen-bond geometry (Å, °)

**Table:**

*D*—H···*A*	*D*—H	H···*A*	*D*···*A*	*D*—H···*A*
O1W—H2W1···O1	0.80 (2)	2.499 (19)	3.0368 (12)	125.8 (16)
O1W—H2W1···O3	0.80 (2)	2.20 (2)	2.9805 (12)	164.7 (18)
O1W—H1W1···O2	0.77 (2)	2.15 (2)	2.8597 (10)	152 (2)
O1W—H1W1···O4	0.77 (2)	2.53 (2)	3.1658 (12)	140 (2)
C8—H8B···O1^i^	0.99	2.42	3.3216 (13)	151
C11—H11A···O1W^ii^	0.95	2.51	3.4242 (13)	161
C5—-H5A···Cg1^i^	0.95	2.88	3.3506 (12)	111
C10—-H10B···Cg1^ii^	0.99	2.73	3.4406 (11)	129
C22—-H22B···Cg2^iii^	0.99	2.87	3.8068 (11)	158

Symmetry codes: (i) −*x*, −*y*, −*z*+2; (ii) −*x*+1, −*y*, −*z*+2; (iii) *x*, *y*+1, *z*.
